# Resveratrol attenuates doxorubicin-induced meiotic failure through inhibiting oxidative stress and apoptosis in mouse oocytes

**DOI:** 10.18632/aging.103061

**Published:** 2020-04-30

**Authors:** Jun Han, Huarong Wang, Tuo Zhang, Ziqi Chen, Ting Zhao, Lin Lin, Guoliang Xia, Chao Wang

**Affiliations:** 1 State Key Laboratory of Agrobiotechnology, College of Biological Sciences, China Agricultural University, Beijing 100193, China; 2 Medical College of Xiamen University, Xiamen 361005, China; 3 Key Laboratory of Ministry of Education for Conservation and Utilization of Special Biological Resources in the Western China, College of Life Science, Ningxia University, Yinchuan 750021, Ningxia, China

**Keywords:** doxorubicin, resveratrol, oocyte maturation, oxidative stress, DNA damage

## Abstract

Doxorubicin (DXR), a widely used chemotherapeutic drug, has adverse effects on female fertility in young cancer patients. However, the underlying mechanisms of doxorubicin exposure on female fertility and how to prevent it have not been well studied yet. Here, mouse oocytes were employed to investigate the issues mentioned above. The results showed that doxorubicin treatment impaired oocyte meiotic maturation by destroying spindle assembly and chromosome arrangement. In addition, doxorubicin caused oxidative stress by increasing reactive oxygen species (ROS) levels. Furthermore, doxorubicin led to severe DNA damage in oocytes, which eventually induced apoptosis through DNA damage-P63-Caspase3 pathway. Conversely, resveratrol (RES) effectively improved oocyte quality by restoring spindle and chromosome configuration, reducing ROS levels and inhibiting apoptosis. In summary, our results indicate that RES can protect oocytes against doxorubicin-induced damage.

## INTRODUCTION

DXR is a widely used anthracycline antibiotic, which is clinically used in the treatment of various malignant cancers, such as leukemia, lymphomas, breast, ovarian and endometrial cancer [1, 2]. However, its clinical use is limited by its dose-dependent toxicity in several organs and systems. Previous studies have shown DXR has cardiotoxicity, hepatotoxicity and reproductive toxicity [3, 4]. Early menopause, premature ovarian failure, and increasing infertility rates are the major problems for young female cancer survivors [5, 6]. In recent years, advances in cancer screening, diagnosis and therapy have markedly improved the survival rate [7]. However, most chemotherapeutic drugs are ovarian toxic. The increasing survival rate of young cancer patients leads to more concern about their fertility state, therefore, it is important to illuminate the toxic effects and possible mechanisms of chemotherapeutic drugs on related organs and cells.

Female mammals are born with a limited number of primordial follicles and the pool of primordial follicles is a non-renewable reproductive resource for animals [8]. Previous studies in humans have shown that chemotherapeutic agents lead to a significant loss of primordial follicles and cause a reduction in ovarian reserve, which consequently shortens the reproductive lifespan of patients [9]. Also, it has been reported *in vivo* that DXR treatment causes significant decrease in the number of primordial, primary and secondary follicles in mice. Furthermore, *in vitro* study in human has demonstrated that DXR accelerates ovarian aging [10, 11]. In ovarian granulosa cells, DXR treatment results in ROS accumulation, mitochondrial membrane potential decrease and apoptosis [12]. DXR exposure causes cell death by DNA damage and cytoplasmic fragmentation in mouse ovulated oocytes [13]. Hence, studies mentioned above suggest that DXR exerts adverse effects on ovary and that is stage and cell-type dependent. Oocyte meiotic maturation is an important event in the female reproduction process. It provides half of the genetic material and maternal components to the embryo, which is crucial for successful fertilization and subsequent embryonic development [14]. However, it remains unclear whether DXR treatment affects oocyte maturation.

RES is a natural phenolic compound and an antimicrobial produced by several plants when they are attacked by pathogens. Accumulating evidence reveals that RES scavenges free radicals and maintains the level of anti-oxidative enzymes to alleviate the damage caused by oxidative stress [15, 16]. Furthermore, it has been reported that RES protects oocyte against postovulatory aging by preventing ROS production. RES also improves the quality of oocytes *in vitro* maturation in mice and humans [17, 18]. Although previous studies have demonstrated the adverse effects of DXR on female germ cell and the protective effects of RES on oocyte maturation, it remains unknown whether RES can protect oocytes against the damage caused by DXR treatment.

In this study, we found that DXR inhibited mouse oocyte meiotic maturation through disturbing spindle assembly and chromosome arrangement. We further demonstrated that DXR caused increased oxidative stress, DNA damage and apoptosis through DNA-damage-P63-Caspase3 pathway in mouse oocytes. On the contrary, RES effectively restored meiotic failure in DXR-treated oocytes, mitigated oxidative stress and rescued the apoptosis caused by DXR treatment.

## RESULTS

### RES alleviates impaired oocyte maturation in DXR-treated oocytes

**Figure 1:**
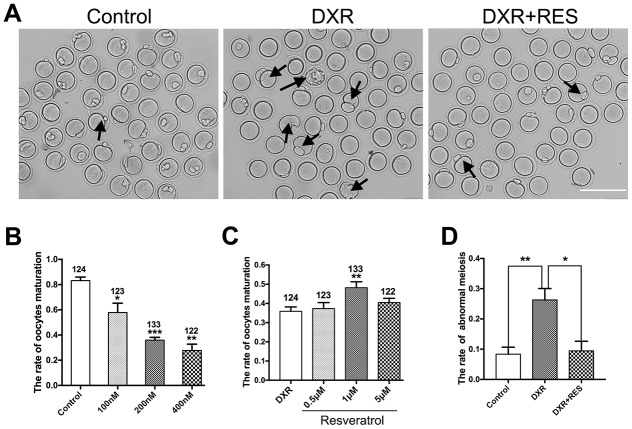
**Effect of different concentrations of DXR and RES on oocyte meiotic maturation.** (**A**) Microscopy images of oocytes morphologies in control, DXR treatment and RES-supplemented group, oocytes exhibited bigger first polar body (black arrowhead) after DXR treatment. Bar = 200 μm. (**B**) The oocyte maturation rate was recorded in control and DXR treatment groups. (**C**) Effect of different concentrations of RES on oocyte maturation in DXR-treated oocytes. The number of oocytes used was shown above the according column. (**D**) The rate of abnormal meiosis was recorded in control, DXR and RES-supplemented oocytes. All experiments were repeated at least 3 times with more than 30 oocytes examined for each experimental condition. Results were represented as means ± SEMs. * means *P* < 0.05, **means *P* < 0.01, *** means *P* < 0.001.

Oocyte meiotic maturation is a unique asymmetric division, producing a large oocyte and a small first polar body. To assess the toxic effects of DXR on oocyte meiotic maturation, we cultured oocytes with increasing concentrations (100 nM, 200 nM, 400 nM) of DXR and calculated the rate of first polar body extrusion, which is the maker of oocyte meiotic maturation. After 12 h culture, as shown in Figure 1A, most oocytes reached the meiosis II (MII) stage (83.21 ± 1.61 %, n = 124) in control group, however, the maturation rate in DXR treatment group significantly decreased, and some oocytes exhibited bigger first polar body, which indicated abnormal cell division. Besides, we observed that when treated oocytes with a higher concentration of DXR (1 μM), the auto-fluorescent of DXR is detectable, accumulating at the chromosome of oocyte, which indicated that DXR directly bound to oocyte chromosome to exert its toxic effects (Supplementary Figure 1). The maturation rate in 400 nM group significantly reduced to 27.63 ± 2.91% (n = 122) (*P* < 0.01), and it was 57.84 ± 4.27% (n = 132) (*P* < 0.05) in 100 nM group and 35.93 ± 1.31% (n = 133) (*P* < 0.001) in 200 nM group (Figure 1B). So, 200 nM was used as the final DXR treatment group in the subsequent experiments.

To examine whether RES could mitigate the impaired oocyte maturation caused by DXR treatment, we cultured oocytes with increasing concentrations of RES (0.5 μM, 1 μM, 5 μM) while treated with 200 nM DXR. As shown in Figure 1, 1 μM RES significantly increased the rate of oocyte maturation (35.93 ± 1.31% *vs.* 48.20 ± 1.78%, *P* < 0.01) and reduced the proportion of abnormal meiotic oocytes comparable to that in control group (8.36 ± 1.34%, n = 97 *vs.* 26.29 ± 1.17%, n = 103, *vs.* 9.46 ± 1.83%, n = 95, *P* < 0.05). The results herein suggested that RES partially alleviated the impaired oocyte maturation caused by DXR.

### RES restores the disrupted spindle structure and chromosome alignment in DXR-treated oocytes

**Figure 2:**
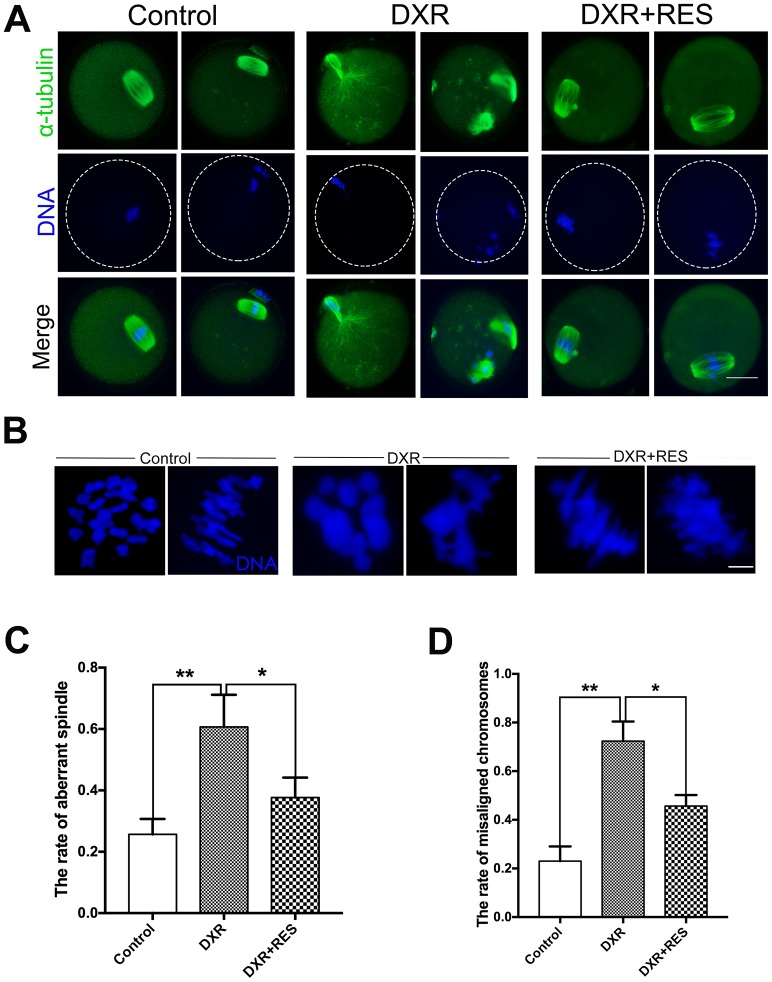
**Effect of RES treatment on spindle morphology and chromosome alignment in DXR-treated oocytes.** Oocytes were immunolabeled with anti-α-tubulin antibody to visualize spindle and counterstained with Hoechst to observe the chromosomes. (**A**) Representative images of spindle morphologies and chromosome alignment in control, DXR-treated and RES-supplemented oocytes. Green, α-tubulin; blue, DNA. Bar = 20 μm. (**B**) Representative images of chromosomes alignment in control, DXR-treated and RES-supplemented oocytes. Blue, DNA. Bar = 5 μm. (**C**) Rate of aberrant spindle was recorded control, DXR-treated and RES-supplemented oocytes. (**D**) Rate of misaligned chromosomes was recorded in control, DXR-treated and RES-supplemented oocytes. All experiments were repeated at least 3 times with more than 30 oocytes examined in each experimental condition. Results were represented as means ± SEMs. *means *P* < 0.05, **means *P* < 0.01.

To investigate the reduced meiotic maturation rate in DXR treatment group, we examined spindle morphology, actin localization and chromosome alignment in both groups. Oocytes were immunolabeled with anti-α-tubulin antibody to label the spindle and counterstained with Hoechst to observe the chromosome. As shown in Figure 2A, 2B, in control group, spindle had the typical barrel shape and chromosome well aligned on the equatorial plate, however, there was no significant difference in actin expression and localization in both groups (Supplementary Figure 2). On the contrary, spindle exhibited irregular configuration, even some aster spindle scattered in DXR treated oocytes, which might be caused by microtubule disassemble. The percentage of oocytes with aberrant spindle morphology (60.67 ± 6.06%, n = 101, *vs.* 25.67 ± 2.91%, n = 102; *P* < 0.01) and misaligned chromosomes (71.67 ± 4.05%, *vs.* 23.00 ± 3.51%, *P* < 0.01) were significantly increased in DXR treated group. After supplement with RES, the abnormal rates were significantly decreased (37.67 ± 3.76%, n = 100, *P* < 0.05, spindle; 45.67 ± 2.6%, n = 101, *P* < 0.05, chromosome), (Figure 2C, 2D). In summary, these results suggested that RES reduced the rate of abnormal meiosis by restoring DXR-induced spindle and chromosome disturbance.

### RES mitigates oxidative stress in DXR-treated oocytes

**Figure 3:**
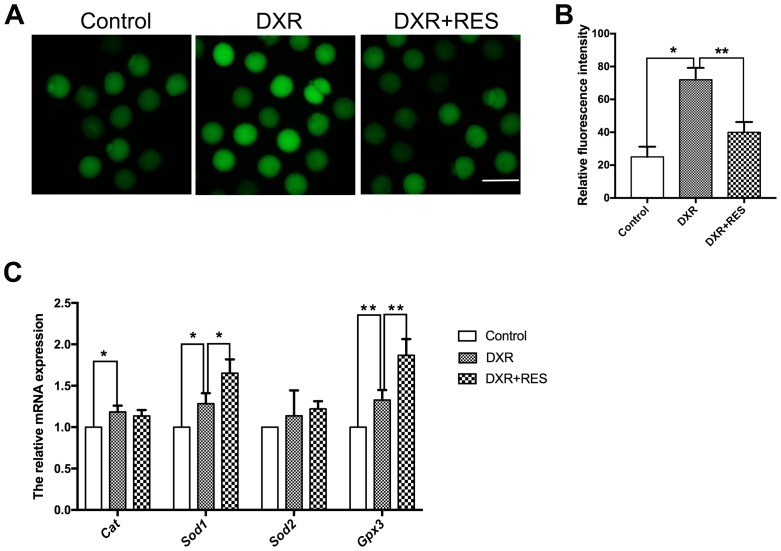
**Effect of RES treatment on ROS levels in DXR-treated oocytes.** (**A**) Representative images showed ROS levels in control, DXR-treated and RES-supplemented oocytes, Bar = 100 μm. (**B**) The fluorescence intensity of ROS was measured in control, DXR-treated and RES-supplemented oocytes. *means *P* < 0.05, **means *P* < 0.01. (**C**) Relative expressions of antioxidant genes *Cat*, *Sod1*, *Sod2* and *Gpx3* mRNA levels in oocytes. β-actin was used as a housekeeping gene. *means *P* < 0.05, **means *P* < 0.01. All experiments were performed in triplicates and the data were represented as the means ± SEM.

Previous studies in different cell lines have demonstrated that ROS is one of the major toxic effects of DXR. To testify whether this was the case in oocytes as well, we detected the ROS level by DCFH-DA fluorescent probe. As shown in Figure 3A, the green signals were considerably stronger in DXR exposure oocytes. Statistical data showed that the fluorescence intensity of ROS was higher in DXR treated group compared with that in control group, but reduced in RES administration group (25 ± 3.61, n = 105 *vs.* 72 ± 4.16, n = 93 *vs* 40 ± 3.61, n = 95, *P*< 0.01) (Figure 3B). We next assessed mRNA expression of antioxidant genes *Cat, Sod1, Sod2* and *Gpx3*. As shown in Figure 3C, the mRNA levels of *Cat*, *Sod1*, and *Gpx3* were significantly increased while the mRNA levels of *Sod2* had no significant difference between the control and DXR treatment groups. Instead, RES reduced ROS levels and elevated the related antioxidant genes expression (Figure 3A–3C). Thus, these results suggested RES prevented oocytes against DXR-induced oxidative stress.

### RES rescues DXR-induced apoptosis by DNA-damage-P63-Caspase3 pathway

**Figure 4:**
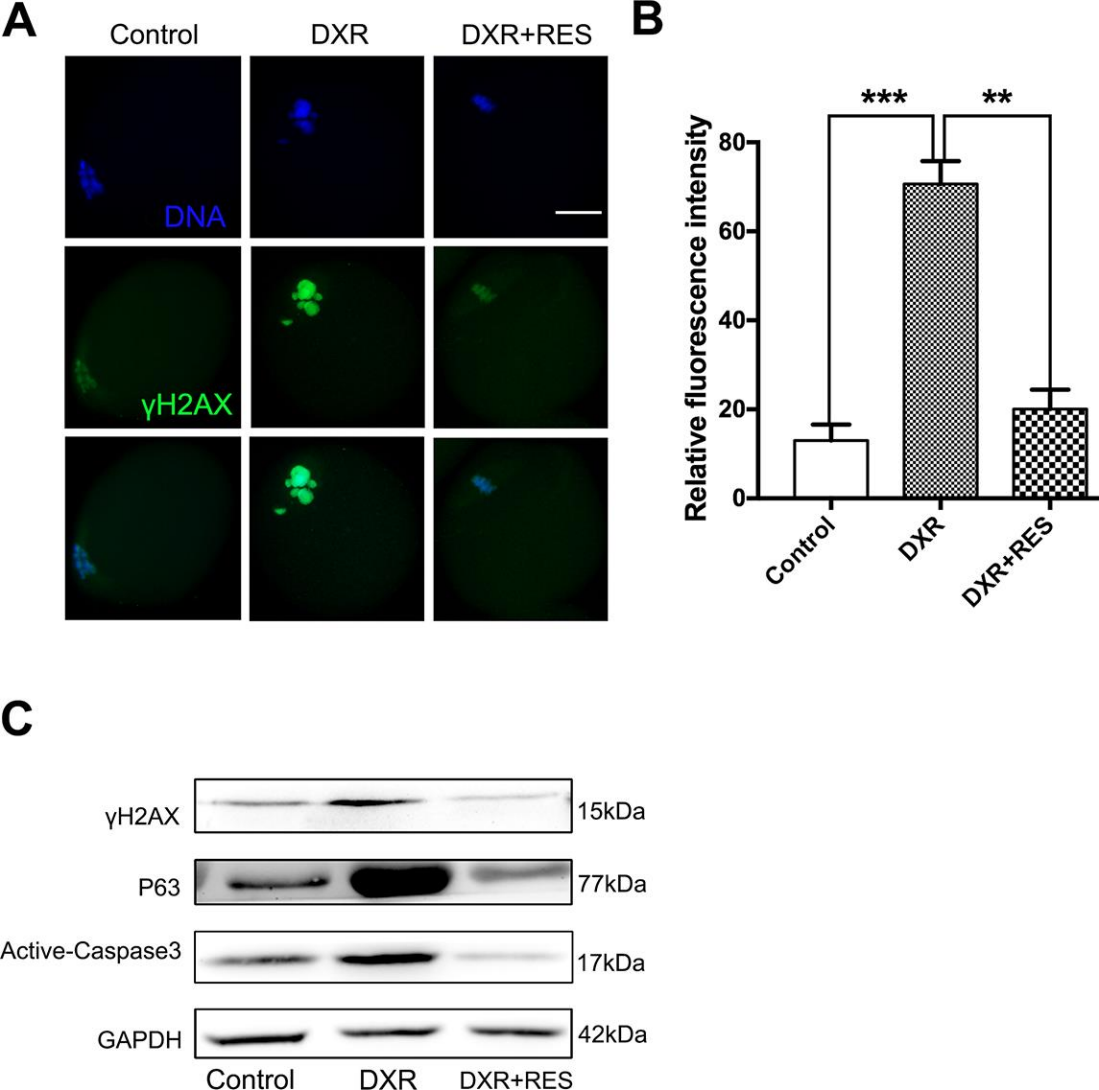
**RES rescued DXR-induced apoptosis through DNA-damage-P63-Caspase3 pathway in mouse oocytes.** (**A**) Representative immunofluorescence images showing the expression of γ-H2AX in mouse oocytes. Green, γ-H2AX, Blue, DNA, Bar = 20 μm. (**B**) The relative immunofluorescence intensity of γ-H2AX was measured in control, DXR-treated and RES-supplemented oocytes. Experiments were repeated at least 3 times with more than 30 oocytes examined for each group. Data were presented as means ± S.E.M of three independent experiments. **means *P* < 0.01, *** means *P* < 0.001. (**C**) Protein levels of γ-H2AX, P63 and Active-Caspase3 were examined by Western blotting in control, DXR-treated and RES-supplemented oocytes. GAPDH was used as a loading control. The clean backgrounds for the active-Caspase-3, γ-H2AX and GAPDH is due to the exposure.

Accumulating evidence has demonstrated that excessive ROS leads to DNA damage. Since DXR treatment caused severe oxidative stress in mouse oocytes, we wonder whether DXR treatment would cause DNA damage to oocytes. H2A histone family member X (H2AX) is one of several genes coding for histone H2A. γ-H2AX is widely used as a marker for DNA DSBs damage. We first examined γ-H2AX expression by immunofluorescence staining. As shown in Figure 4A, 4B, DXR treatment caused severe DNA damage to oocytes, whereas RES reduced DNA damage signal to an indistinguishable level compared with that in control group according to quantitative analysis of fluorescence intensity (70.67 ± 2.96, n = 95 *vs.* 13.00 ± 2.08, n = 102 *vs* 20.00 ± 2.52, n = 93, *P* <0.05). To further explore the possible pathway responsible for DXR-induced DNA damage, we then examined the protein expression of P63. Previous studies in ovarian follicles demonstrated that P63 was involved in cisplatin-induced DNA damage in mouse oocytes [19]. As shown in Figure 4C, P63 expression was markedly increased in DXR treated oocytes. Since DNA damage often triggered apoptotic pathways, we next examined active Caspase3 in both groups. As shown in Figure 4C, compared with control group, active Caspase3 expression was significantly increased in DXR-treated group, which indicated DXR treatment induced cell death in oocytes. As expected, RES rescued DXR-induced apoptosis by DNA-damage-P63-Caspase3 pathway (Figure 4A–4C).

## DISCUSSION

Compromised fertility is one of the side effects of chemotherapy. Therefore, it is imperative to explore the molecular mechanisms of DXR treatment on female reproductive performance to take effective prevention methods in the clinic. Recent studies have demonstrated that RES protects oocytes from chemical agents-induced oxidative stress and apoptosis. Thus, we designed this study to investigate the possible protective role of RES on DXR-induced damage in mouse oocytes.

First, our results confirmed that DXR treatment inhibited oocyte maturation. We noticed that although some DXR-treated oocytes extruded first polar body, they exhibited bigger polar body compared with oocytes in the control group, which indicated that DXR caused abnormal cell division in mouse oocytes. Previous has shown that the enlarged first polar body is associated with impaired fertilization and embryo quality [20]. By contrast, when supplemented with RES, oocytes abnormal meiosis rate was significantly decreased. Microtubules and actin filaments are the main sources of mechanical force during oocyte meiotic maturation. DXR treatment disrupted spindle configuration and chromosome alignment while DXR treatment did not affect microfilament expression, which was similar to previous *in vivo* study using follicle as a model [21]. Meanwhile, RES administration partially restored the disrupted spindle configuration and chromosome alignment in DXR-treated oocytes. Therefore, we suggested that RES alleviated the impaired oocyte maturation caused by DXR treatment.

Several studies have shown that DXR-induced oxidative stress is one of the primary mechanisms for its toxic effects on noncancerous tissues and cells [22–24]. In physiological conditions, ROS generated during the metabolic process is a signaling molecule in female reproductive process. However, oxidative stress occurs when the equilibrium between ROS and antioxidants is disrupted [25]. In our study, after DXR treatment, ROS levels were significantly increased, which was similar to previous studies in cardiomyocyte [3, 26]. Our results also revealed that DXR upregulated the mRNA expression of antioxidant genes, which possibly due to the self-protect mechanism of oocytes. That is, oocytes may try to correct the imbalance of oxidative stress state but the increase may not high enough to resist the increase of ROS levels. Previous studies have demonstrated that elevated ROS levels disrupt spindle structure and chromosome arrangement in mouse oocytes, resulting in mismatched chromosome and causing seriously damage on oocyte quality [27, 28]. Thus, we speculated that the disorganized spindle and chromosome aberrations we observed in DXR-treated oocytes might be caused by oxidative stress, which would ultimately inhibit oocyte maturation and reduce oocyte developmental competence.

RES is an effective antioxidant regulating the related antioxidant enzymes to protect cell against oxidative damage [15]. Notably, our results showed that RES significantly reduced ROS levels, increased the expression of antioxidant enzymes genes and alleviated the oxidative stress caused by DXR. However, RES failed to rescue the mRNA expression of *Gpx3*. The possible reason was that RES protected oocytes from DXR-induced oxidative stress by improving mitochondrial function or increasing the activity of antioxidant enzymes as reported in previous studies [29, 30]. Thus, we demonstrated that RES could improve oocyte quality through reducing oxidative damage.

DXR administration causes DNA damage in different cell types in mouse ovary and human primordial follicle [11, 13, 31], which suggests that DNA damage might be a universal mechanism responsible for its toxic effects. Consistent with these studies, our results also showed that DNA damage was markedly increased after DXR treatment. P63 plays essential roles in mediating DNA damage-induced cell death during meiotic arrest and P63 is activated when oocytes are exposed to irradiation or chemotherapeutic drug cisplatin in primordial follicles [19, 32, 33]. Our results showed that P63 expression was increased significantly after DXR treatment, which was in agreement with former studies in primordial follicles. Besides, it is well documented that extensive DNA damage triggers apoptosis in the context of DXR-induced ovarian injury [13, 31, 34]. In accordance with these studies, DXR led to increased active-Caspase3 expression in oocytes in this study. In contrast, RES attenuated DXR-induced damage through DNA-damage-P63-Caspase3 pathway.

In summary, this study shows that RES restores meiotic failure in DXR-treated oocytes by reducing ROS levels and apoptosis. These findings contribute in several ways to our understanding of DXR-induced injury to oocyte maturation and might be assistance to female fertility preservation in the clinic. However, the safety and effectiveness of RES need further investigation.

## MATERIALS AND METHODS

### Animals

CD-1 female mice (21-23 days old) were purchased from the Laboratory Animal Center of the Institute of Genetics and Developmental Biology (Beijing, China). All procedures were approved by the Animal Care and Use Committee of China Agricultural University and were performed following the Animal Research Institute Committee guidelines. Mice were raised in a temperature and light-controlled room, water and food were feed *ad libitum*.

### Oocyte collection and culture

Immature female CD-1 mice were killed by cervical dislocation to collect ovaries. Germinal vesicle intact oocytes were picked with a pipetted tube, each group contains at least 30 oocytes, washed several times in M2 medium, then cultured under mineral oil in M16 medium h at 37°C in 5% CO_2_ atmosphere.

### Reagents and antibodies

Doxorubicin hydrochloride and resveratrol were purchased from J&K Chemical Ltd. (Shanghai, China). Throughout this paper, the abbreviation DXR and RES were used to refer to doxorubicin hydrochloride and resveratrol respectively. DXR and RES were dissolved in DMSO, then diluted to the corresponding working concentrations, with the final concentration of DMSO not more than 0.1% of the culture medium. Concentrations range of doxorubicin in the study were determined from the relevant plasma level (50-200 nM) in cancer patients [35]. Finally, 200 nM was chosen as the final dosage in the following experiments.

Mouse anti-α-tubulin-FITC antibody (#F2168) was purchased Sigma-Aldrich (MA, USA); rabbit polyclonal anti-γ-H2AX antibody (#NB100-2280) was purchased from Novus (CO, USA), rabbit polyclonal anti-P63 antibody (#BS1279) was purchased from Bioworld (MO, USA), Rabbit polyclonal anti-active-Caspase3 antibody was purchased from Beyotime, (Shanghai, China). Alexa Fluor 488 goat anti-rabbit antibody, Alexa Fluor 594 goat anti-mouse antibody were from Invitrogen (CA, USA).

### Immunofluorescence

Oocytes were fixed 30 min in 4% paraformaldehyde, then permeabilized with 0.5% Triton X-100 for 20 min, and blocked 1 h in blocking buffer (PBS containing 1% BSA) at room temperature, oocytes were then probed with different primary antibodies (1:400 for anti-α-tubulin-FITC; 5 μg/ml Phalloidin-TRITC; 1:200 for γ-H2AX) at 4°C overnight, after extensive washing, oocytes were then stained with the corresponding secondary antibodies. Finally, oocytes were stained with Hoechst for 10 min to visualize the DNA, then transfer oocytes to a glass slide. The slides were examined and photographed using Nikon Eclipse 80i digital fluorescence microscopy.

The fluorescence intensity was assessed using Image J software (NIH). Samples from different groups were mounted on the same glass slide and scanned with the same parameters of fluorescence microscopy.

### Analysis of intracellular ROS generation

Intracellular ROS levels were measured by detecting ROS as green fluorescent signals of DCFH diacetate (DCHFDA; Beyotime Biotechnology, Shanghai, China). After 9 h culture, oocytes from both groups were incubated at 37 °C for 30 min in D-PBS that contained 10 μM DCHFDA. Then, the oocytes were washed 3 times with D-PBS and placed on glass slides and covered with coverslips. After that, each sample was observed immediately under a Nikon fluorescent microscope with the same scan settings.

### Western blotting analysis

At least 100 oocytes were collected in each group, dissolved in SDS sample buffer then boiled at 100°C for 10 min. Protein lysates were separated by SDS-PAGE and electrophoretically transferred to PVDF membranes (Millipore, MA, USA). The membranes were blocked in Tris-buffered saline containing 0.1% Tween 20 and 5% no fat dry milk for 1 hour, then probed with primary antibodies, overnight at 4°C. After several times washing, the membranes were incubated with a horseradish peroxidase-linked secondary antibody. Finally, bands on the membranes were detected using the Super-Signal West Pico chemiluminescent detection system (Prod 34080, Thermo, USA).

### RNA extraction and quantitative real-time RT-PCR

RNA was extracted from at least 150 oocytes for different groups using Trizol Reagent according to the manufacturer’s protocol. Quantitative PCR was performed on a Light Cycler instrument (Roche, Mannheim, Germany). Gene expression changes were analyzed by the 2^-ΔΔ^Ct method and normalized with β-actin. The primers used for testing genes are listed in Supplementary Table 1.

### Statistical analysis

Each experiment repeated at least 3 times with independent samples. Data are presented as means ± SEMs and analyzed by one-way ANOVA. *P* value < 0.05 was considered significant.

## Supplementary Material

Supplementary Figures

Supplementary Table 1
