# Supplementary material for: Resveratrol attenuates doxorubicin-induced meiotic failure through inhibiting oxidative stress and apoptosis in mouse oocytes
Source: Aging (Albany NY). 2020 Apr 30;12(9):7717–28. doi: 10.18632/aging.103061 (PMC7244048; doi:10.18632/aging.103061)
Supplement: Supplementary Figures [file aging-12-103061-s001..pdf]

## SUPPLEMENTARY FIGURES

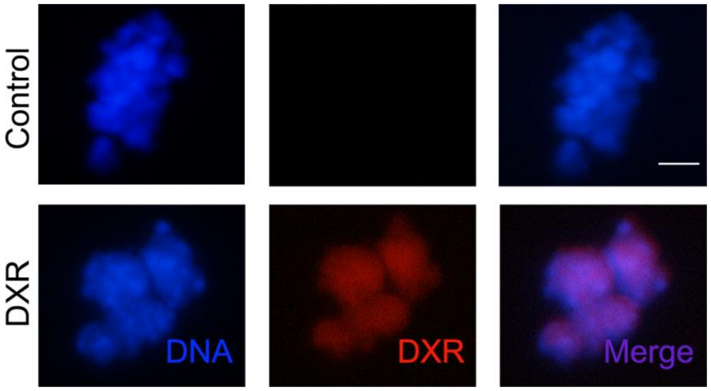


**Supplementary Figure 1. Co-localization of DXR and oocyte chromosomes.** DXR-treated oocytes showed positive fluorescent signal and co-localized with DNA in oocytes. Blue, DNA; red, DXR.

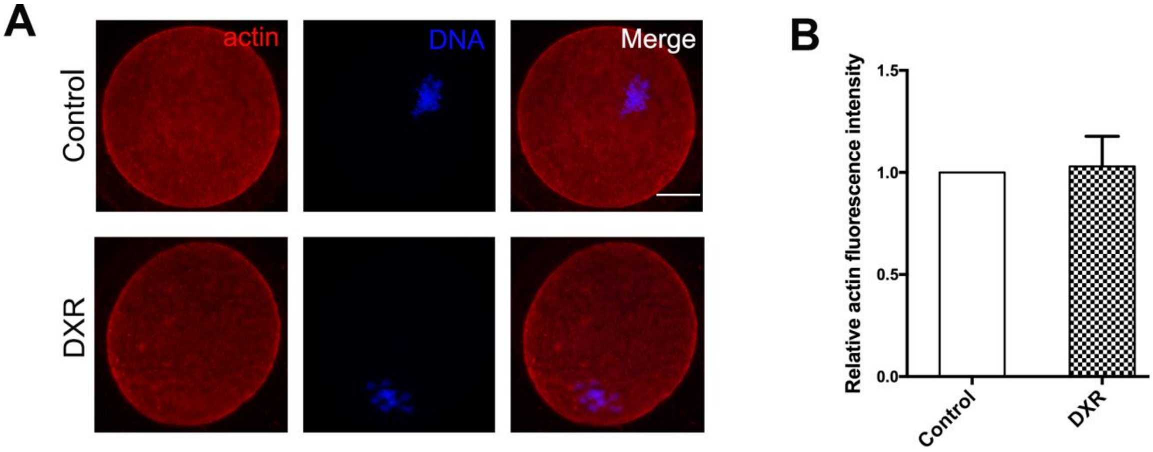


**Supplementary Figure 2. DXR exposure did not affect actin expression in mouse oocytes.** (**A**) Representative images of actin localization in control and DXR-treated oocytes. (**B**) The fluorescence intensity of actin was measured in control and DXR-treated oocytes. Results were presented as means ± SEMs of at least 3 independent experiments with more than 30 oocytes examined for group. Bar = 20 µm.
