# Supplementary material for: Resveratrol attenuates doxorubicin-induced meiotic failure through inhibiting oxidative stress and apoptosis in mouse oocytes
Source: Aging (Albany NY). 2020 Apr 30;12(9):7717–28. doi: 10.18632/aging.103061 (PMC7244048; doi:10.18632/aging.103061)
Supplement: Supplementary Table 1 [file aging-12-103061-s002..pdf]

SUPPLEMENTARY TABLE

Supplementary Table 1. Primer sequences for qRT-PCR.

	Forward	Reverse
Cat	ACCAAATACTCCAAGGCAAAGGT	CAAACCCACGAGGGTCCCGA
Sod1	GCTGTACCAGTGCAGGTCCTCA	CATTTCCACCTTTGCCCAAGTC
Sod2	AAAGCGGTGTGCGTGCTGAA	CAGGTCTCCAACATGCCTCT
Gpx3	CCTCAAGTACGTCCGACCTG	CAATGTCGTTGCGGCACACC
β-actin	TTGTTACCAACTGGGACG	GGCATAGAGGTCTTTACGG
